# Staufen1 promotes HCV replication by inhibiting protein kinase R and transporting viral RNA to the site of translation and replication in the cells

**DOI:** 10.1093/nar/gkw312

**Published:** 2016-04-22

**Authors:** Updesh Dixit, Ashutosh K. Pandey, Priya Mishra, Amitabha Sengupta, Virendra N. Pandey

**Affiliations:** Department of Microbiology, Biochemistry and Molecular Genetics, New Jersey Medical School, Rutgers, Rutgers, the State University of New Jersey, 185 South Orange Avenue, Newark, NJ 07103, USA

## Abstract

Persistent hepatitis C virus (HCV) infection leads to chronic hepatitis C (CHC), which often progresses to liver cirrhosis (LC) and hepatocellular carcinoma (HCC). The molecular mechanisms that establish CHC and cause its subsequent development into LC and HCC are poorly understood. We have identified a cytoplasmic double-stranded RNA binding protein, Stau1, which is crucial for HCV replication. In this study, Stau1 specifically interacted with the variable-stem-loop region in the 3′ NTR and domain IIId of the HCV-IRES in the 5′ NTR, and promoted HCV replication and translation. Stau1 coimmunoprecipitates HCV NS5B and a cell factor, protein kinase R (PKR), which is critical for interferon-induced cellular antiviral and antiproliferative responses. Like Stau1, PKR displayed binding specificity to domain IIId of HCV-IRES. Stau1 binds to PKR and strongly inhibits PKR-autophosphorylation. We demonstrated that the transport of HCV RNA on the polysomes is Stau1-dependent, being mainly localized in the monosome fractions when Stau1 is downregulated and exclusively localized in the polysomes when Stau1 is overexpressed. Our findings suggest that HCV may appropriate Stau1 to its advantage to prevent PKR-mediated inhibition of eIF2α, which is required for the synthesis of HCV proteins for translocation of viral RNA genome to the polysomes for efficient translation and replication.

## INTRODUCTION

Approximately 3% of the world's population is estimated to be infected with HCV (1), the major cause of chronic liver disease. Some infected individuals can clear the virus without treatment. However, most infections persist if untreated, leading to chronic hepatitis C (CHC), which may further lead to liver cirrhosis and hepatocellular carcinoma (2). Although persistent viral infection of liver cells is a major factor in the onset and development of CHC, the role of specific cell factors in promoting chronic HCV infection is not clearly understood. The HCV genome is a positive-stranded RNA with conserved and highly structured untranslated 5′ and 3′ terminal regions, which have multiple regulatory elements that are essential for viral replication and translation. Various cell factors have been reported to interact with 5′ NTR and 3′ NTR (3–7). Recently, we designed a novel strategy to capture replicating HCV RNA genome *in situ* and have identified many cell factors associated with the viral genome (8). One of these factors is Staufen1 (Stau1), which we have earlier shown to be crucial for HCV replication (8). Blackham and McGarvey have also shown that Stau1 is required for efficient HCV replication and production of virion particles (9).

Stau1 was originally identified in *Drosophila melanogaster*, in which it is required for specific localization of maternal RNA in the *Drosophila* egg (10). A human homolog of Stau1 has also been identified and characterized (11,12). Stau1 is a multifunctional double-stranded RNA (dsRNA)-binding cell protein (13). It is involved in embryonic stem-cell differentiation (14), mRNA transport and localization (15,16), mRNA translational activation (17) and Staufen-mediated decay (SMD) of mRNA (18–22). It is overexpressed in HIV-1-infected cells and incorporated into packaged HIV-1 virions (23,24). Stau1 interacts with the NS1 protein of influenza virus and is essential for viral replication (25). It also interacts with an ATP-dependent RNA helicase (UPF1) and triggers translation-dependent degradation of specific mRNA upstream of the Stau1-binding-site (SBS). We found that Stau1 is required for HCV replication; its downregulation nearly abolishes HCV replication in Huh7.5 cells (8). The molecular mechanism of Stau1-mediated regulation of HCV replication and associated pathogenesis is not known.

Various eukaryotic proteins specifically interact with dsRNA species to regulate signaling events and gene expression in cells. Double-stranded RNA binding proteins (DRBPs) residing in the nucleus mainly function in RNA interference (RNAi), mRNA elongation, editing, stability, splicing and export, whereas cytoplasmic DRBPs function in the regulation of translation, dsRNA signaling events and host defense (26). Stau1 is a cytoplasmic DRBP that interacts with PKR and may be involved in preventing a PKR-mediated translational shutdown in cells. PKR functions in host defense against virus infection. Many of the viral RNAs are capable of activating PKR, which inhibits translation via eIF2α phosphorylation. Many other viruses have devised mechanisms to inhibit PKR, thus preventing the inhibition of protein synthesis, which would be detrimental to their replication (27). In the present study, we have explored the mechanism whereby HCV modulates the function of Stau1 to its advantage and prevents a PKR-mediated shutdown of specific cell factors that may inhibit HCV replication.

## MATERIALS AND METHODS

### Plasmids, oligonucleotides and antibodies

Plasmids pLMH14 and pMH14, respectively carrying the HCV subgenomic replicon with and without the luciferase reporter gene, were obtained from Makoto Hijikata (Kyoto University, Japan). Plasmids pVP506 and pVP709 respectively containing the coding region of HCV-3′ NTR and HCV 5′ NTR, were constructed by cloning their PCR-amplified fragment between NdeI and BamHI sites in the pET3b vector (Novagen). Plasmid pGFP-h-Stau1 was a gift from Matsumi Hirose (University of Tokyo, Japan); pET28a-Stau1 was constructed by cloning PCR-amplified Stau1 coding region between *BamH1* and *HindIII* sites. A bicistronic reporter plasmid, pGEM-REN-HCV IRES-Luc, containing renilla and firefly luciferase, was a gift from Dr. Fanxiu Zhu (Florida, USA) (28). Plasmid pPET- PKR/λPP was purchased from Addgene, (Cambridge, USA) for the expression of unphosphorylated PKR. Plasmid pGFP-h-PKR was a gift from Dr. Bin Tian (Rutgers University-Newark, USA);

A mixture of three Stau1-siRNAs (SC-76586) was purchased from Santa Cruz Biotechnology (Dallas, TX, USA). These were SC-76586A sense: CGA GUA AAG CCU AGA AUC Att, antisense, 5′-UGA UUC UAG GCU UUA CUC Gtt-3′; SC-76586B, sense:5′-CUG AGC AAC UGG ACU AUC Utt-3′, antisense 5′-AGA UAG UCC AGU UGC UCA Gtt-3′; and SC-76586C, sense:5′-CUA CAC UAC AGG AUA UGA Utt-3′, antisense, 5′-AUC AUA UCC UGU AGU GUA Gtt-3′. The control siRNA with scramble sequence (5′- UUC UCC GAA CGU GUC ACG Utt-3′, antisense 5′-ACG UGA CAC GUU CGG AGA Att-3′) was also obtained from Santa Cruz. Primers used for RT-PCR of HCV 5′ NTR, GAPDH, and actin mRNAs, as well as for PCR amplification of full-length HCV 3′ NTR and 5′ NTR and their specific fragments were purchased from Sigma-Aldrich (St. Louis, USA). Primary antibodies against HCV NS5B, Staufen1 and protein kinase PKR were obtained from Santa Cruz Biotechnology.

### Cell culture, preparation of cell lysates, preparation of nuclear and cytoplasmic extract, immunoprecipitation, western blotting and construction of stably transduced cells knocked down for targeted proteins

Huh7.5 cells, MH14 cells with replicating HCV subgenomic replicons, and cured MH14 cells were maintained as previously described (29,30). Both MH14 and cured MH14 cells are derived from the Huh7 hepatoma cell line. The transfection of cells with siRNA, preparation of cell lysates and nuclear and cytoplasmic extracts, co-immunoprecipitation and western blotting were done as described earlier (31).

### *In-vitro* transcription of the full-length HCV subgenomic replicon, HCV 3′ and 5′ NTR RNA, and HCV fragments

*In vitro* transcription of full-length HCV subgenomic replicon RNA, as well as Cy5-labeled full-length HCV 3′- and 5′-NTR and their fragments, was as described earlier (31). The transcripts were purified by phenol-chloroform extraction and ethanol precipitation, dissolved in DEPC-treated water and stored at −80°C.

### Photoaffinity crosslinking of HCV 3′- and 5′-NTR with Stau1

We incubated 500 ng of purified Stau1 with Cy5-labeled HCV 3′-NTR or 5′-NTR in the presence or absence of unlabeled RNA competitor corresponding to 3′- or 5′-NTR. The incubation buffer contained 50 mM Tris HCl (pH7.8), 1 mM DTT, 1 mM MgCl_2_ and 0.01% bovine serum albumin in a final volume of 20–30 μl. After 20 min of incubation on ice, we UV irradiated the mixture (Spectrolinker XL-1000; Spectronics at 360 mJ/cm^2^. We treated the irradiated samples with RNase A (0.1 μg/μl; Qiagen, (Valencia, CA, USA) for 15 min at 37°C and resolved the crosslinked RNA–protein complexes on 8% sodium dodecyl sulfate (SDS) polyacrylamide gel. The labeled RNA–protein complex was visualized using a Typhoon scanner (Amersham). To confirm the Stau1-RNA crosslinked complex in the cell lysate, we transferred the complex onto a nitrocellulose membrane (Schleicher and Schuell Bioscience, (New Hampshire, USA) and western blotted for Stau1 using anti-Stau1 antibody (SC-134042, Santa Cruz Biotechnology).

### Photoaffinity crosslinking of PKR with HCV 5′ NTR

Crosslinking of PKR with 0.5 pmol of Cy5-labeled full-length HCV 5′-NTR was done in the absence or presence of *in vitro* transcribed cold RNA fragments corresponding to domains 1 & II, IIIabc, IIId and IV of HCV 5′-NTR. The incubation buffer contained 50 mM Tris HCl (pH7.8), 1 mM DTT, 1 mM MgCl2, 0.01% bovine serum albumin and 2–5 μg of purified PKR in a final volume of 20 μl. After 20-min incubation on ice, we UV-irradiated the mixture in a Spectrolinker at 360 mJ/cm^2^. To map domain-specific binding of PKR, we used internally ^32^P-labeled *in-vitro*-transcribed RNA fragments for photo-crosslinking with PKR. The crosslinked complexes were treated with RNase A, resolved by SDS-PAGE and visualized using a phosphorImager.

### Quantification of HCV RNA in the cell by quantitative real-time RT-PCR

We used 1 μg of total cellular RNA to synthesize cDNA corresponding to HCV 5′ NTR and GAPDH mRNA by reverse transcription (31). We used 50 ng of cDNA to do quantitative real-time PCR (Fast Real PCR System, Applied Biosystems, as we have described (32,33). Data were analyzed using either 7500 software (Applied Biosystems) or by using ΔΔCT method. The relative changes in RNA copies of HCV were calculated by normalizing the amount of GAPDH mRNA present in each sample. For each point of qPCR data, statistical significance *P* value was obtained by using two-tailed unpaired *t*-test with Graph Pad software. All experiments were performed in triplicate for each data point.

### Quantification of the gel image

All gel images (western blotting and RT-PCR) were quantified by either QuantityOne software (version 4.4.1; Bio-Rad) or ImageJ software. All analyses were based on triplicate experiments. The statistical significance *P*-value was obtained for each band by using two-tailed unpaired *t*-test with Graph Pad software.

### Luciferase assay

Cells in 24-well plates (3 × 10^4^/well) were first transfected with Stau1 siRNA (final concentration, 20 nM/well) or Stau1-overexpressing plasmid pGFP-hStau1 (3 μg per well), using Lipofectamine 2000 (Invitrogen) according to the manufacturer's protocol. At 24-h post-transfection, cells were transfected with HCV replicon RNA carrying luciferase reporter (0.5 μg/well) and 100 ng of a pRL-luc plasmid expressing Renilla luciferase (2) under the control of SV40 promoter. Cells were grown for another 48 h, lysed and assayed for luciferase using a dual luciferase reporter assay kit (Promega). Assays were performed in four parallel sets.

### Polyribosome fractionation and northern blotting

To examine the effect of Stau1 on HCV translation, we did polyribosome binding assays in Huh7.5 cells in which Stau1 was either downregulated or overexpressed. We first transfected the Huh7.5 cells with Stau1 siRNA or Stau1 overexpression plasmid. Twenty-four hours later, cells were transfected with HCV-R-IRES-Luc plasmid (pGEM-R-H-L) with Lipofectamine 2000. After 48 h, cells were lysed and processed for isolation of polysomes fraction by ultracentrifugation on sucrose density gradient as described earlier (34,35). Fractions of 500 μl were collected from the bottom of each tube. Each fraction was treated with proteinase-K (125 μg /ml) in a solution containing 0.2 M Tris-HCl (pH7.5), 25 Mm EDTA, 0.3 M NaCl, 2% SDS and 5 units of RNase-free DNase-l. We then isolated total RNA from each fraction and subjected it to northern blot analysis. To examine the level of HCV–IRES mRNA in polysome fractions, northern blots were incubated overnight at 42ºC with an 18-mer Cy3-labeled DNA probe complementary to HCV 5′-NTR (Cy3-5′-AGT ACC ACA AGG CCT TTC G-3′), washed with (0.1X SSC -0.1% SDS) at 37ºC and visualized in a Typhoon scanner.

#### Expression and purification of recombinant PKR

Recombinant clone of pPET- PKR/λPP was expressed in *E. coli* Rosetta (DE3) and purified by Hi-Trap Heparin column (Pharmacia). In brief, transformed *E. coli* Rosetta (DE3) cells were grown at 37°C in Luria Broth (LB) medium containing 100 μg/ml of ampicillin until an OD_595_ of 0.4 was achieved. The medium was cooled to 25°C, supplemented with 1 mM IPTG and further incubated at 25°C for 16 h with vigorous shaking. The cells were harvested, washed and resuspended in a lysis buffer containing 20 mM Tris-HCl, pH 7.4; 200 mM NaCl; 1 mM β-mercaptoethanol; 10% glycerol; 1% Triton-X 100; and 1× ProteoBlock protease inhibitor cocktail (Fermentas) containing 2 mg/ml lysozyme. The suspension was sonicated and centrifuged. The clear supernatant was applied to a Hi-Trap Heparin column pre-equilibrated with binding buffer (20 mM Tris-HCl, pH 7.4; 200 mM NaCl; 10% glycerol). The column was washed extensively, and PKR was eluted with a linear gradient (0–80%) of 1 M KCl in the same buffer for 20 min (1 ml/min). Eluted fractions showing more than 95% purity on SDS-PAGE (8%) were pooled and dialyzed against buffer containing 50 mM Tris-HCl (pH 7.5), 2 mM DTT, 100 mM NaCl and 50% glycerol.

#### Expression and purification of recombinant Stau1, Auf1 and HIV Tat

Recombinant clone of His-tagged Stau1 (pET28a-Stau1), Auf1 (pET28a-Auf1) and HIV Tat (pET23a-HIV Tat) were expressed in *E. coli* Rosetta (DE3) and purified by affinity chromatography using Ni-NTA and Hi-Trap Heparin columns (Pharmacia). In brief, transformed *E. coli* Rosetta (DE3) cells were grown at 37°C in Luria Broth (LB) containing 30 μg/ml of kanamycin (for clones in pET28a vector) or 100 μg/ml ampicillin (for clone in pET23a vector) until an OD_595_ of 0.5 was achieved. The medium was supplemented with 1.0 mM IPTG, and further incubated at 37°C for 4 h with vigorous shaking. The cells were harvested, washed and resuspended in a lysis buffer containing 20 mM Tris-HCl, pH 7.4; 200 mM NaCl; 1 mM β-mercaptoethanol; 10% glycerol; 1% Triton-X 100; 5 mM imidazole; and 1× ProteoBlock protease inhibitor cocktail (Fermentas) containing 2 mg/ml lysozyme. The suspension was sonicated and centrifuged. The clear supernatant was applied to a Ni-NTA column pre-equilibrated with binding buffer (20 mM Tris-HCl, pH 7.4; 200 mM NaCl; 10% glycerol and 5 mM imidazole). The column was washed with the binding buffer containing 50 mM of imidazole. The bound proteins were then eluted with 200 mM imidazole in the same buffer. Eluted fractions showing more than 95% purity on SDS-PAGE were pooled and dialyzed against buffer containing 50 mM Tris-HCl (pH 7.5), 2 mM DTT, 100 mM NaCl and 50% glycerol.

### PKR autophosphorylation

We used pPET- PKR/λPP (Addgene, Cambridge, MA, USA) for expression of unphosphorylated PKR (36). The plasmid pPET- PKR/λPP expresses both PKR and bacteriophage λ protein phosphatase (λPPase) genes located downstream of the T7 promoter. The PKR autophosphorylation assay was carried out under standard conditions as described earlier (37,38). In brief, reactions were carried out in a final volume of 20 μl containing 20 mM Tris HCl (pH7.5), 5 mM MgCl_2_, 5 mM MnCl_2_, 100 mM KCl, 2 μM MATP, 5 μCi γ-^32^P ATP, 0.1 mM EDTA, 0.1 μg poly(I).poly (C), 30% (v/v) glycerol and variable concentrations of unphosphorylated PKR. The reaction, carried out for 30 min at 30ºC, was terminated by the addition of 1× Laemmli sample buffer. Samples were heated for 5 min at 95ºC, then resolved by 10% SDS PAGE. Phosphorylated PKR was visualized by phosphorimaging.

#### Dephosphorylation of PKR protein

Dephosphorylation of PKR was done by using calf intestinal alkaline phosphatase (CIAP, cat# 18009–027, Invitrogen). For dephosphorylation, we added 1 unit CIAP per microgram of PKR protein in CIAP dephosphorylation buffer (Invitrogen) and incubated for different time points at 37°C. To stop the reaction, we added 1× protein loading dye and resolved on an 8% SDS-polyacrylamide gel.

#### Immunoprecipitation

MH14 or Huh7.5 cells (5 × 10^6^ cells per assay) were washed twice with warmed 1× PBS and then lysed in cold buffer containing 50 mM Tris–HCl (pH 7.4), 150 mM NaCl, 1% Triton X-100 and 1× protease inhibitor cocktail (Roche Applied Science). We also treated the lysates with 50 units of the benzonase (Sigma) to avoid nonspecific RNA/DNA binding proteins that might be captured in immunoprecipitation (IP) via RNA/DNA bridging. We incubated the cell lysates with 2 μg of primary antibody against the target protein for 1 h at 4°C, then added 20 μl of protein A/G Plus agarose beads (Santa Cruz, Biotechnology). The mixture was then incubated overnight at 4°C on a rotary mixture. The immunoprecipitates were collected by centrifugation at 2500 rpm for 5 min at 4°C. After washing the pellets four times with lysis buffer, we resuspended the immunoprecipitates in 1× Laemmli gel loading buffer. Samples were boiled and centrifuged to pellet the agarose beads. The supernatant was subjected to SDS-PAGE, and western blotted for the target proteins.

#### Competition between Stau1 and PKR for binding to domain IIId of HCV IRES

We first incubated a fixed concentration (1 pmol) of Stau1 or PKR with 1 pmol *in-vitro*-transcribed Cy5-labeled domain IIId RNA on ice for 15 min and then supplemented with increasing concentration (1–3 pmol) of the competitor (PKR or Stau1) protein. After 20-min incubation on ice, the mixture was photocrosslinked, treated with RNase-A and resolved by SDS-PAGE.

### RNA-dependent RNA polymerase (RdRp) assay

The RdRp assay was done in a final volume of 100 μl. The reaction mixture contained 50 mM Tris HCl (pH 7.8), 100 mM NaCl, 5.0 mM DTT, 0.01% bovine serum albumin (BSA), 0.01% Tween-20, 5% glycerol, 100 μM cold UTP, 1.5 μCi/assay ^3^H UTP, 500 nM Poly rA/dT_18_ and 100 nM HCV NS5B enzyme. The enzyme was preincubated in the reaction buffer along with 100 nM Stau1 at room temperature for 15 min, after which polymerase reactions were initiated by the addition of 1.0 mM MnCl_2_ to the reaction mixture. After 30 min of incubation at 37°C, reactions were terminated by the addition of ice-cold 5% TCA and samples were kept on ice for 15 min. The acid-precipitable nucleic acid material was filtered on glass fiber filters (GF/B), washed successively with 5% TCA, water and ethanol. Filters were air dried and placed in a vial containing 5 ml EcoLite scintillation fluid, then counted for radioactivity using a Packard 2200-CA Tri-Carb scintillation counter.

### Immunofluorescence

We incubated 1 × 10^5^ MH14 cells per well on BD Falcon 8-chamber tissue culture slides and grew them for 24 h. We washed the cells and processed them for immunofluorescence using primary antibodies against Stau1, PKR or NS5B as described (31). The slides were mounted with mounting medium (prolong® Antifade Kit, Molecular Probes, Eugene, OR, USA) and visualized under a confocal microscope (Nikon Air) using NIS software. To determine the extent of co-localization, we used Nikon NIS viewer analysis software to calculate the overlap coefficient between 0 and 1 range (0 is minimum, and 1 is maximum co-localization).

## RESULTS

### Mapping of the Stau1 binding site on HCV 3′ NTR

**Figure 1.**
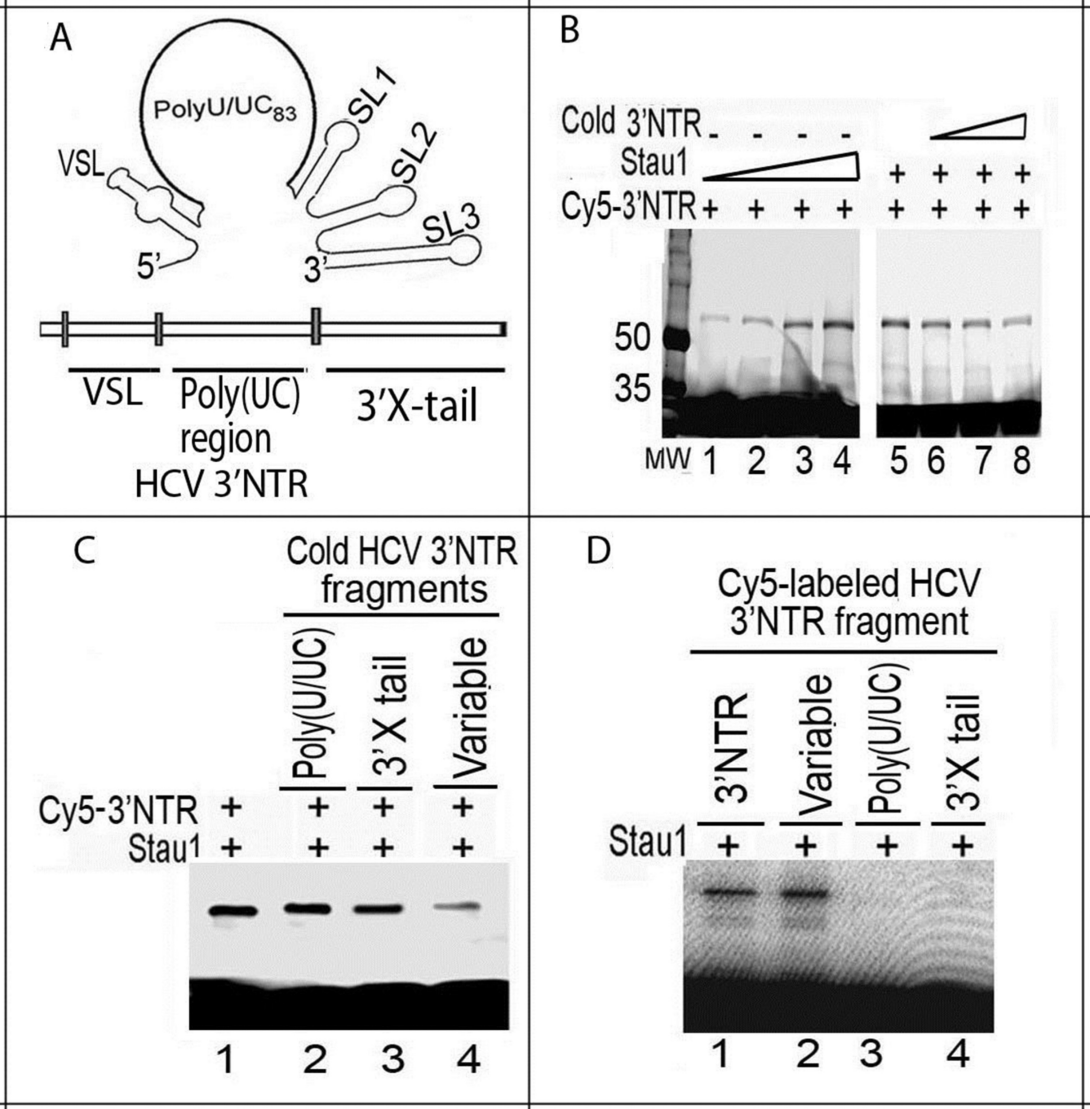
Mapping of interaction between Stau1 and HCV 3′NTR. (**A**) The structure of 3′NTR showing VSL, the poly-(UC)-rich region and the conserved 3′X-tail (68) (**B**) Crosslinking of Cy5-labeled HCV 3′NTR with Stau1 as a function of increasing Stau1 concentration. Cy5-labeled 3′NTR (0.5 pmol) was incubated with 0.25, 0.5, 1.0 and 1.25 pmol of purified Stau1 (lanes 1 through 4, respectively). In lanes 5 through 8, a fixed 1 pmol concentration of Stau1 was crosslinked with a fixed 1 pmol concentration of Cy5-labeled HCV 3′NTR in the presence, respectively, of 0.5, 1, 2.5 and 5 pmol of cold HCV 3′ NTR. (**C**) Stau1 binding to 3′NTR is specifically out-competed by RNA fragments corresponding to the VSL region. One pmol of Stau1 was crosslinked with 0.5 pmol of Cy5-labeled full-length 3′NTR in the absence (lane 1) or presence of *in-vitro*-transcribed cold RNA fragments corresponding to the poly (UC) rich region (lane 2), conserved 3′X-tail (lane 3) and VSL region (lane 4). (**D**) Stau1 superficially binds to VSL region in the 3′NTR. Stau1 (1 pmol) was crosslinked with 0.5 pmol of Cy5-labeled RNA fragments corresponding to full-length 3′ NTR (lane 1), VSL (lane 2), poly (U/UC) (lane 3) and 3′X-tail (lane 4).

Previously, we captured Stau1 as a cell factor that binds to HCV 3′ NTR (39). Recently, we affinity-captured replicating HCV subgenomic replicon *in-situ* and identified Stau1 as one of the cell proteins associated with the viral genome (8). In this study, we mapped the interaction between Stau1 and 3′ NTR and identified the structural motif of 3′ NTR specifically recognized by Stau1. We did UV-mediated photoaffinity crosslinking of Cy5-labeled *in-vitro*-transcribed 3′ NTRs with increasing concentrations of purified recombinant Stau1 protein. The crosslinked RNA–protein complex was treated with RNase-A (0.1 μg/μl), then resolved on an 8% SDS-polyacrylamide gel. We found that Stau1 efficiently photo-crosslinks with HCV 3′ NTR (Figure 1B, lanes 1–4), which is effectively out-competed by cold unlabeled 3′ NTR (Figure 1B, lanes 6–8). To identify the specific structural motif of HCV 3′ NTR (Figure 1A) with which Stau1 interacts, we did competition experiments with *in-vitro* transcribed unlabeled RNA fragments corresponding to the poly (UC) rich region, conserved 3′ × tail region, and variable stem-loop (VSL) region of 3′ NTR. We found that binding of Stau1 to full-length Cy5-labeled 3′ NTR RNA (Figure 1C, lane 1) is out-competed by the RNA fragment corresponding only to the VSL region of 3′ NTR (lane 4), but not by the conserved 3′ X-tail (lane 3) or poly (UC) regions (lane 2). We confirmed that Stau1 specifically binds to the VSL region in the 3′ NTR by photo-crosslinking with Cy5-labeled VSL, poly (U/UC), and 3′ X-tail (Figure 1D, lane 2). These findings suggest that Stau1 specifically binds with the variable region of HCV 3′ NTR that is required for HCV replication (40).

### Mapping of the Stau1 binding site on HCV 5′ NTR

**Figure 2.**
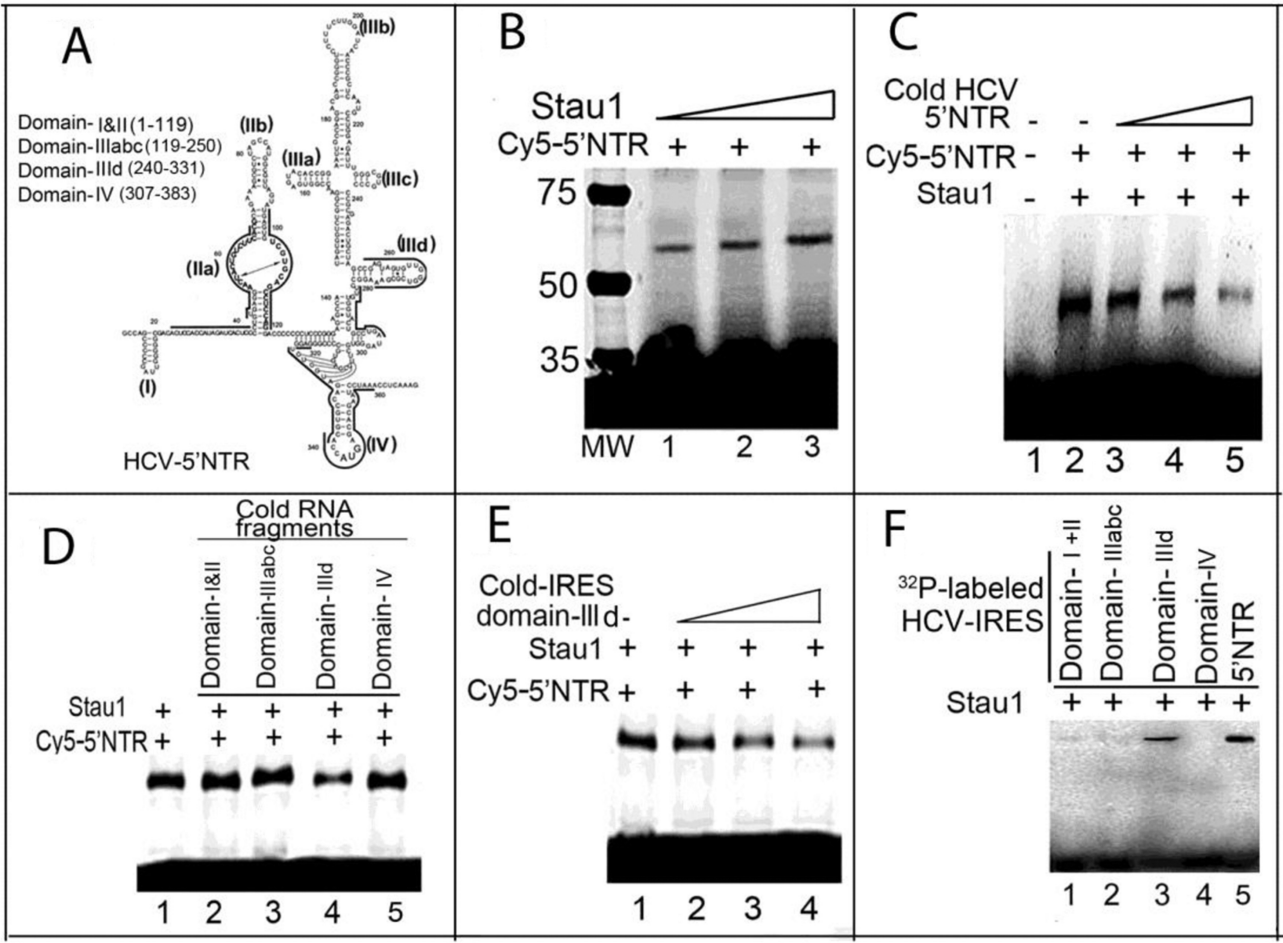
Mapping of interaction between Stau1 and HCV 5′NTR. (**A**) The predicted the secondary and tertiary structure of 5′NTR (69). (**B**) UV-mediated photo-crosslinking of Cy5-labeled HCV 5′NTR. We incubated 0.5 pmol of Cy5-labeled HCV 5′NTR with increasing concentrations (0.5, 0.75 and one pmol) of purified Stau1 (lanes 1–3). The samples were UV-irradiated, treated with RNaseA and resolved by SDS-PAGE. Crosslinked RNA–protein complexes were detected with a Typhoon scanner. (**C**) Binding of Stau1 with Cy5-labeled HCV 5′NTR is out-competed by cold HCV 5′NTR. Lane 1, 5′NTR alone; Lanes 2–5, 1 pmol of Stau1 crosslinked, respectively, with 0.5 pmol of Cy5-labeled 5′NTR in the absence and presence of 0.5, 2.5 and 5 pmol of cold HCV 5′NTR. (**D, E**) Binding to Stau1 with 5′NTR is specifically out-competed by domain IIId of HCV IRES. (**F**) Stau1 specifically binds to RNA fragments corresponding to domain IIId of 5′NTR. Lanes 1–5, Stau1 photo-crosslinked with 32P-labeled RNA fragments corresponding, respectively, to domains I+II, IIIabc, IIId and IV of HCV 5′NTR. Lane 5, ^32^P-labeled full-length 5′NTR RNA.

We examined whether Stau1 also interacts with HCV 5′ NTR. We photo-crosslinked *in-vitro*-transcribed Cy5-labeled full-length HCV 5′ NTR with increasing concentrations of purified recombinant Stau1, finding that Stau1 efficiently binds and crosslinks with Cy5-labeled 5′ NTR (Figure 2B), which can be out-competed by unlabeled 5′ NTR RNA (Figure 2C, lanes 3–5). We further mapped the structural motif of 5′ NTR (Figure 2A), which specifically recognized by Stau1. We photo-crosslinked Stau1 with Cy5-labeled 5′ NTR in the absence or presence of unlabeled RNA fragments corresponding to domains I and II, IIIabc, IIId and IV of the 5′ NTR (Figure 2D). We found that Stau1 binding to full-length 5′ NTR is specifically out-competed by domain IIId of the HCV IRES in the 5′ NTR (Figure 2D, lane 4; Figure 2E, lanes 2–4). By photo-crosslinking Stau1 with ^32^P-labeled individual structural domains of 5′ NTR, we confirmed that Stau1 specifically recognizes domain IIId (Figure 2F, lane 3).

### Downregulation of Stau1 severely impairs HCV replication

**Figure 3.**
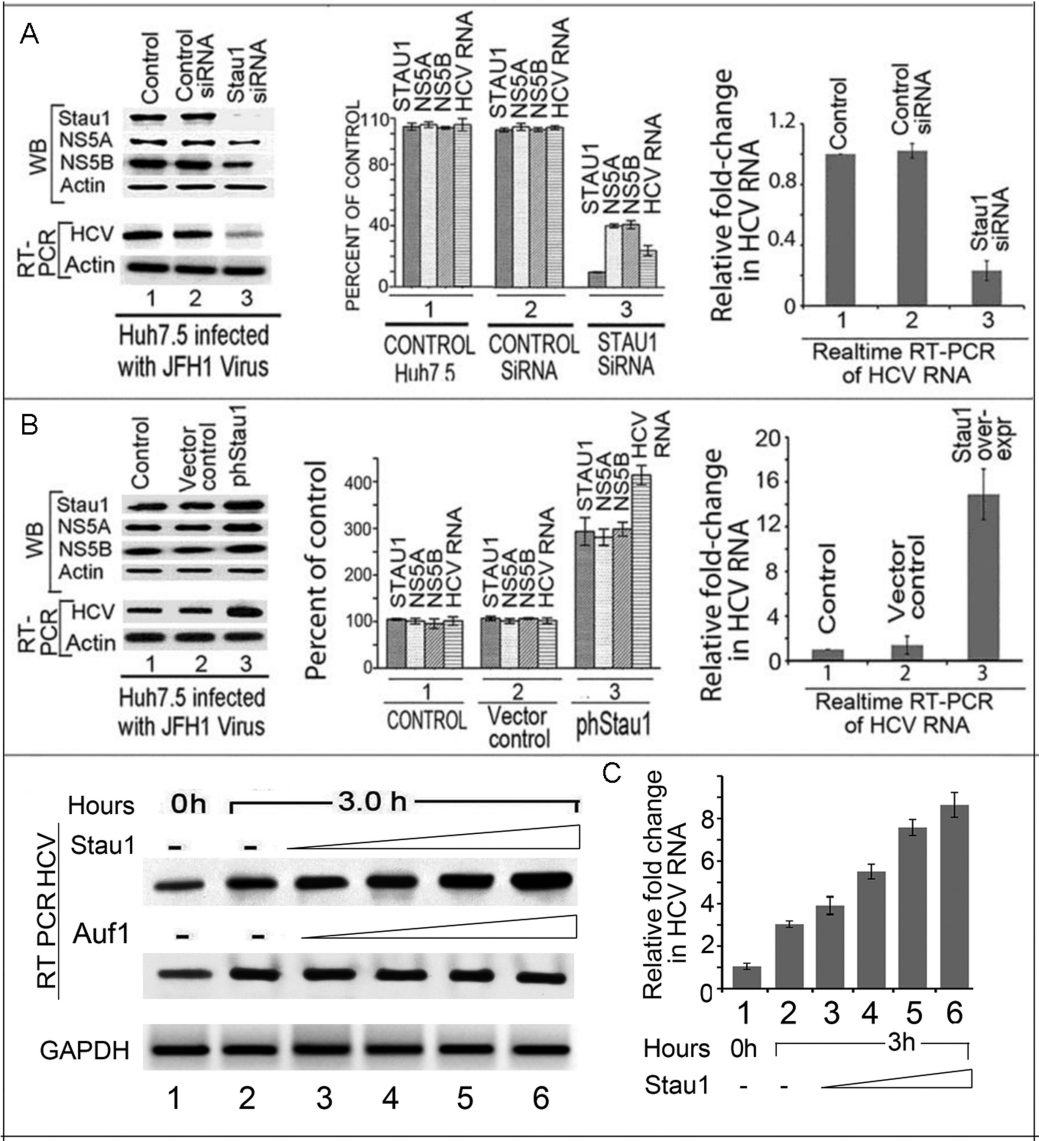
Effect of Stau1 on HCV replication and translation. (**A**) Downregulation of Stau1 inhibits HCV replication and translation. Huh7.5 cells transfected with Stau1 siRNA or control siRNA were grown for 24 h, then infected with JFH1 HCV. Forty-eight hours later, cell lysates were prepared, and western blotted for Stau1, NS5A, NS5B and RT-PCR on total RNA to determine levels of HCV RNA and GAPDH mRNA. Lane 1, untransfected controls; Lane 2, cells transfected with control siRNA; Lane 3, cells transfected with Stau1 siRNA. The middle panel shows quantitation of WB and RT-PCR bands. The right panel shows quantitative RT PCR of HCV RNA and GAPDH mRNA. Relative fold change in HCV RNA was calculated by normalizing the amount of GAPDH mRNA in each sample. All experiments were done in triplicate for each data point. (**B**) Overexpression of Stau1 enhances HCV replication and translation. Huh7.5 cells transfected with overexpressing Stau1 clone (**lane 3**) or vector alone (**lane 2**) were grown for 24 h, then infected with JFH1 HCV. Forty-eight hours later, cells were analyzed for the expression of Stau1, NS5A, NS5B and actin by WB. Total RNA was isolated from another set of experiments and analyzed for HCV RNA and GAPDH mRNA by RT-PCR. Lane 1, control; Lane 2, vector control; Lane 3, Stau1 overexpressed. The middle panel shows the quantitation of WB and RT-PCR bands. The right panel shows quantitative RT PCR of HCV RNA. (**C**) Effect of exogenously added Stau1 on *in-vitro* endogenous HCV replication in cell-free replication lysate. Endogenous HCV replication of cell-free replication lysate was done as described in the Materials and Methods (41,70). Lanes 1 and 2, control replication lysate alone incubated at 0 and 3 h, respectively. Lane 3–6 cell-free replication lysate supplemented with increasing concentration (0.25–1.5 pmol) of either Stau1 (top panel) or Auf1 (middle panel).

Earlier, we demonstrated for the first time that downregulation of Stau1 strongly inhibits replication of HCV subgenomic replicons in MH14 cells (8). Subsequently, Blackham and McGivney (9) have also shown that downregulation of Stau1 by three different siRNAs against Stau1 significantly reduced the level of both HCV RNA and viral proteins. In this study, we used a cocktail mixture of three Stau1-siRNAs (SC-76586) to examine the effect of Stau1-downregulation on the replication of infectious JFH1 HCV in Huh7.5 cells. We found that siRNA-mediated downregulation of Stau1 severely impaired HCV replication in Huh7.5 cells (Figure 3A, left panel, lane 3). As compared to untransfected controls (lane 1) or cells transfected with scrambled control siRNA (lane 2), the levels of viral proteins NS5A and NS5B were also drastically reduced in cells transfected with Stau1-siRNA (lane 3). Quantitative real-time PCR of HCV RNA indicated a 5-fold reduction of HCV RNA in Stau1-siRNA-treated cells as compared to untransfected control or control siRNA transfected cells (Figure 3A, right panel, lane 3). These results indicate that Stau1 is a crucial cell factor that is required for efficient HCV replication.

### Upregulation of Stau1 enhances HCV replication and translation of viral proteins

Since downregulation of Stau1 drastically reduced HCV replication in Huh7.5 cells, we examined the effect of its overexpression on HCV replication and translation. We first transfected pGFP-hStau1 in Huh7.5 cells and then infected with JFH1 HCV. Forty-eight hours later the levels of Stau1 and viral proteins were determined by WB and HCV RNA by quantitative real-time PCR (Figure 3B). We found that overexpression of Stau1 significantly enhanced the HCV replication and translation of viral proteins NS5A and NS5B (Figure 3B, left panel lane 3) as compared to untransfected control and vector control (lanes 1, 2). The quantitative RT-PCR indicated a 10-fold increase in HCV RNA level in Stau1-overexpressing cells (Figure 3B, right panel, lane 3) as compared to the level in controls (lanes 1 and 2), confirming that Stau1 promotes both HCV replication and translation of viral proteins.

### Exogenously added Stau1 stimulates *in-vitro* endogenous HCV replication in the cell-free replicative lysate of MH14 cells

Having found that overexpression of Stau1 significantly enhances HCV replication in Huh7.5 cells, we examined whether *in-vitro* endogenous HCV replication in a cell-free replication system is stimulated in the presence of exogenously added purified Stau1. We prepared cell-free replication lysate from MH14 cells (31,41) and applied it to a cell-free HCV replication assay in the absence or presence or exogenous Stau1 (Figure 3C). We included AUF1, an RNA binding protein, as control which promotes HCV translation (7) but does not have any effect on HCV replication. After 3 h of incubation, we isolated the total RNA and examined the level of HCV RNA by RT-PCR and qPCR, which reflected the activity of endogenous HCV replicative complexes in the replicative lysate. In the presence of exogenous Stau1, the level of newly synthesized HCV RNA was 3- to 6-fold higher in the presence of increasing concentration (0.25–1.5 pmol) of Stau1 (lanes 3–6) as compared to the basal level of replication activity in the absence of Stau1 (lane 2). In contrast, we found no change in the endogenous replication in the presence of exogenously added Auf1 (lanes 3–6). AUF1 is an AU-rich element (ARE)-binding proteins, which we have earlier identified as one of the HCV 3′-NTR binders (39) which also interacts with stem-loop II of HCV IRES and promotes HCV translation (7). These results indicate that Stau1 is positively involved in modulating the HCV replication complex.

### Stau1 is essential for HCV translation

**Figure 4.**
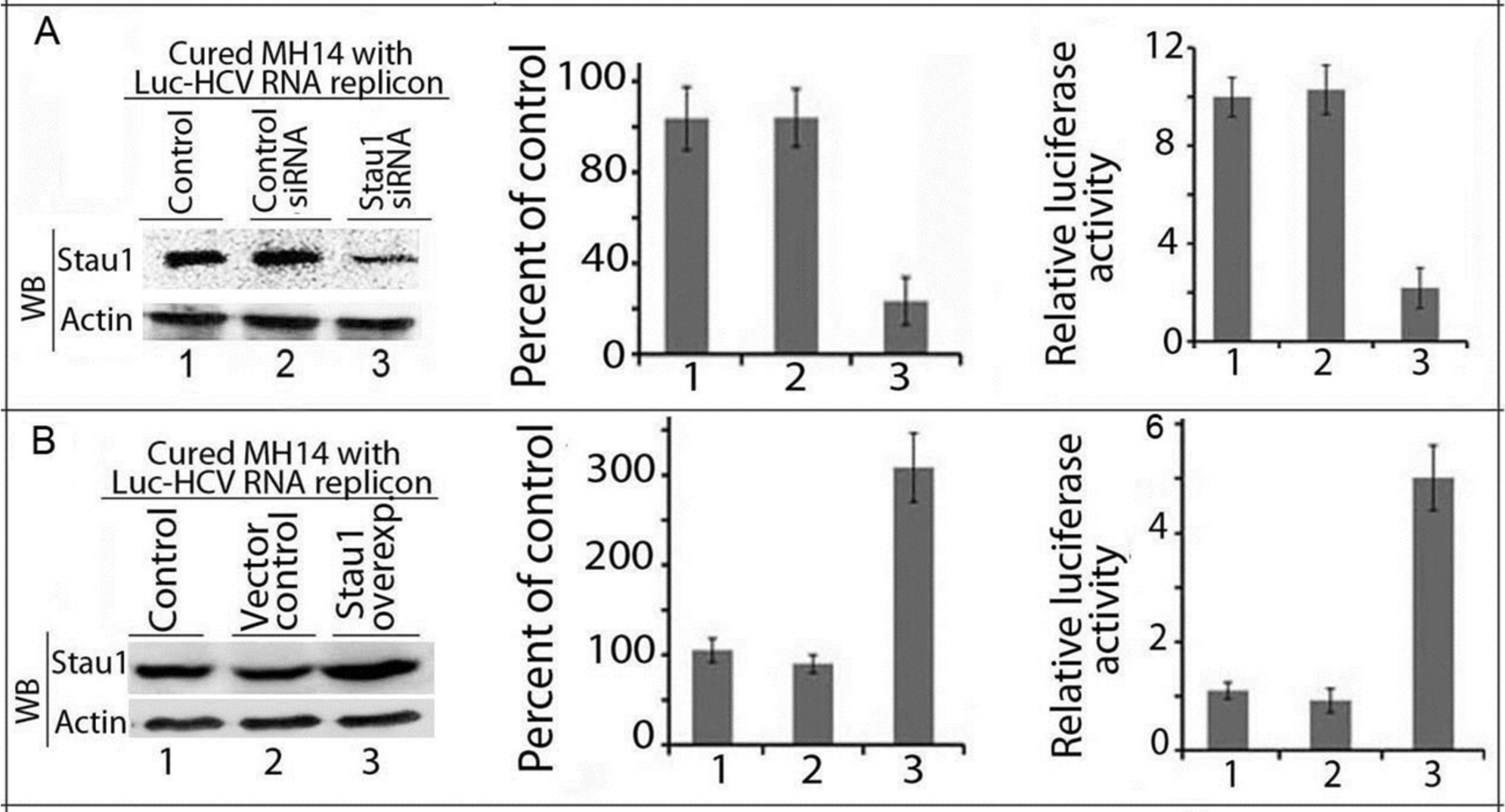
Stau1 is essential for HCV translation. Cured MH14 cells without HCV replicons were transfected with Stau1 siRNA (Figure 4A) or Stau1 overexpressing plasmid (Figure 4B); 24 h later, cells were co-transfected with reporter Luc-HCV-replicon RNA and pRL-SV40 expressing renilla luciferase. Forty-eight hours later, cell lysates were western blotted for Stau1 and actin (left panel) and assayed for dual luciferase activity (right panel). Lane 1, control; lane 2, siRNA control; lane 3, Stau1 downregulated or overexpressed. The middle panel shows the quantitation of each WB band expressed as percent of control.

Since Stau1 specifically interacts with domain IIId of HCV IRES, we examined whether downregulation of Stau1 influences the translation mediated by HCV IRES. We used Luc-HCV-RNA replicon, in which firefly luciferase is placed downstream of HCV 5′ NTR. We co-transfected Luc-HCV RNA and pRL-Luc in cured MH14 cells in which Stau1 was either downregulated by transfecting Stau1-siRNA or overexpressed by pGFP-hStau1. We measured luciferase activity in the cell lysate 48 h later, and western blotted for the expression of Stau1. We found a 4-fold decrease in the expression of Stau1 in cells treated with Stau1-siRNA (Figure 4A, left and middle panels, lane 3) with a concomitant 5-fold reduction in the relative reporter activity (Figure 4A, right panel, lane 3). In contrast, transfection of cells with pGFP-hStau1 resulted in a 3-fold increase in Stau1 expression (Figure 4B, left and middle panels lane 3) and a 5-fold increase in the reporter activity (Figure 4B, right panel, lane 3). These findings suggest that Stau1 may be important in HCV translation as well.

### Stau1 is required for transport of HCV RNA to polyribosomes

**Figure 5.**
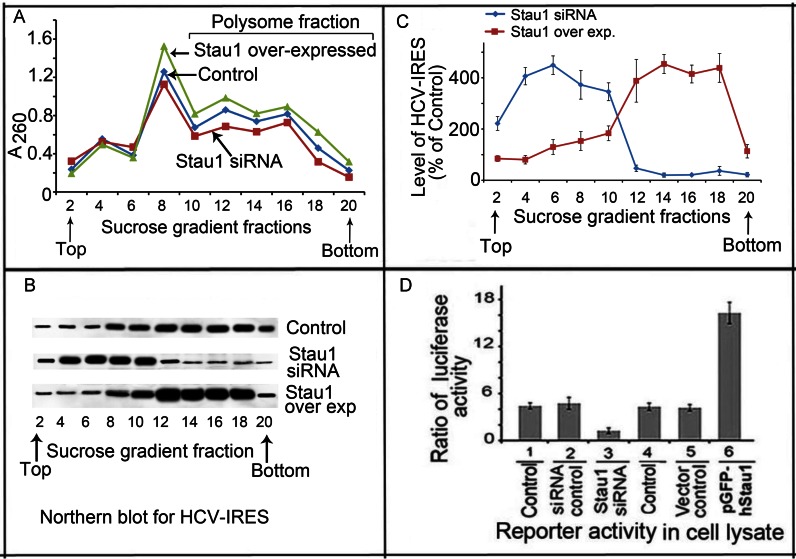
Stau1 is required for transfer and loading of HCV RNA onto polysomes. (**A**) Profile of OD^260^ of sucrose density fractions. Control Huh7.5 cells and cells in which Stau1 was either downregulated or overexpressed were transfected with pHCV-IRES; after 48 h, cells were lysed and processed for polysome profiling by ultracentrifugation on a sucrose density gradient (34,35). (**B**) Northern blot analysis of HCV-IRES level in the alternate gradient faction. (**C**) Quantitation of the HCV-IRES level in an alternate fraction as a percent of control in cells in which Stau1 was either overexpressed (orange) or downregulated (25). (**D**) Stau1 is required for HCV IRES-mediated translation. We co-transfected pHCV-IRES-Luc and pRL-SV40 in Huh7.5 cells in which Stau1 had been either downregulated or overexpressed. Lanes 1 and 4, control; lane 2, control siRNA; lane 3, Stau1-siRNA; lane 5, vector control; lane 6, cells transfected with pGFP-hStau1 (Stau1 overexpression). After 48 h, luciferase activity in the cell lysate was measured.

Since Stau1 is involved in transport and localization of cellular mRNAs (15,16), we examined whether it also facilitates transport and loading of HCV RNA to polysomes. We transfected pHCV-IRES-Luc in Huh7.5 cells in which Stau1 was either downregulated or overexpressed and, 48 h later, lysed cells and processed them for isolation of polysome fractions by ultracentrifugation on a sucrose density gradient (34,35). We collected fractions from the bottom and measured the OD^260^ of each fraction to identify polysomes-containing fractions (Figure 5A). Total RNA from each fraction was isolated, and northern blotted to determine the level of HCV-IRES, using a Cy5-labeled probe complementary to HCV-IRES (Figure 5B). We found high levels of HCV-IRES containing reporter mRNA concentrated in polysomes fractions #12–18 in control cells, as well as in cells in which Stau1 was overexpressed. In contrast, HCV IRES remained in the monosome-containing fractions #4–10 in cells in which Stau1 was downregulated. Quantitation of HCV IRES RNA in each fraction demonstrated 400% increase in the polysomes fractions of Stau1-overexpressing cells but was negligible in cells treated with Stau1-siRNA (Figure 5C).

A parallel set of cells was co-transfected with pHCV-IRES-Luc and pRL-SV40 reporter plasmids; 48 h later, cell lysates were measured for luciferase activity using a dual luciferase reporter assay kit. Results (Figure 5D) indicated that reporter activity directed by HCV IRES was drastically reduced in Stau1-downregulated cells (lane 3) but significantly enhanced on upregulation of Stau1 (lane 6). These results clearly establish the role of Stau1 as an essential cell factor, not only for HCV replication but also for transport and loading of HCV-IRES onto polysomes for translation.

### Stau1-IP co-immunoprecipitates HCV NS5B and cell factor PKR

**Figure 6.**
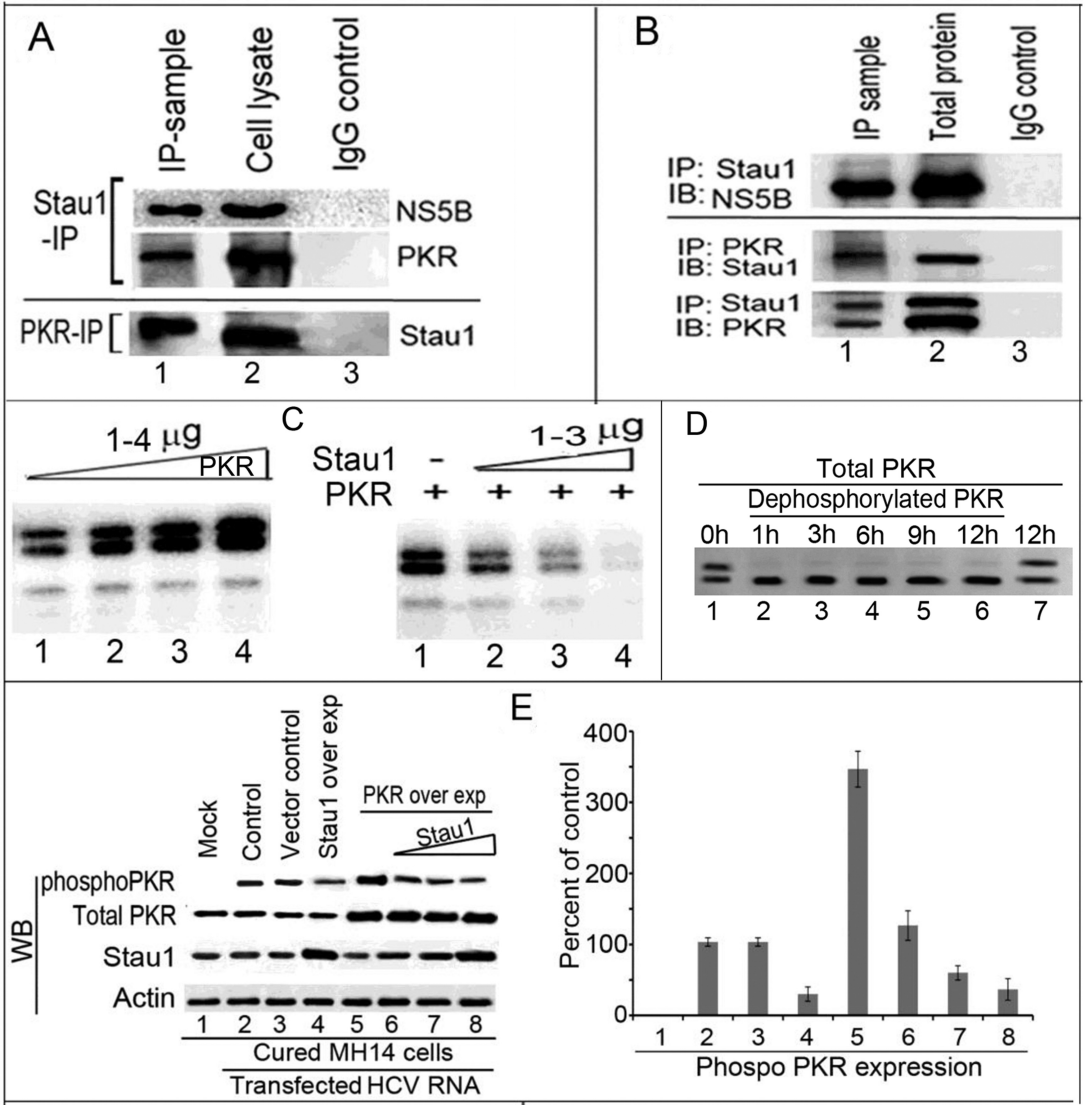
Stau1 interacts with PKR and NS5B and inhibits PKR-autophosphorylation. (**A**) Upper panel: Stau1-IP coprecipitates NS5B and PKR. Stau1-IP on cell lysate of JFH1 HCV-infected Huh7.5 cells co-precipitates NS5B and cell factor PKR. Lane 1, Stau1-IP; Lane 2, cell lysate; lane 3, IgG isotype control. **Lower panel:** PKR-IP co-precipitates Stau1. Lane 1, PKR-IP; Lane 2, cell lysate; lane 3, IgG control. (**B**) Upper panel: Immunoblot of Stau1-IP on a mixture of recombinant purified Stau1+ NS5B. Lane1, Stau1 IP was immunoblotted for NS5B; lane 2, total protein; lane 3, IgG control. **Lower panel:** Reciprocal IP of Stau1 and PKR on a mixture of purified Stau1+PKR. Lane 1, IP sample immunoblotted for either Stau1 or PKR; lane 2, total protein; lane 3, IgG control. (**C**) Autophosphorylation of PKR is inhibited by Stau1. Left panel shows autophosphorylation of PKR as a function of increasing concentration of PKR in the absence of Stau1 (lanes 1–4). The increasing concentrations of purified recombinant PKR were incubated with 2 μM γ-^32^P-ATP (5 μCi) in the phosphorylation buffer as described before (37,38). Right panel shows inhibition of PKR autophosphorylation in the presence of Stau1. Two micrograms of purified recombinant PKR were incubated in the phosphorylation buffer in the absence (lane 1) or presence of increasing concentrations of Stau1 (lanes 2–4). (**D**) Dephosphorylation of recombinant PKR. We treated 1 μg of purified recombinant PKR with 1 unit of Calf intestinal alkaline phosphatase (CIAP) at 37°C for 1–12 h in the dephosphorylation buffer and then resolved by SDS PAGE. Lanes 1 and 7, untreated PKR control incubated for 0 h and 12 h, respectively; Lanes 2 through 6, recombinant PKR treated with CIAP for 1, 3, 6,9 and 12 h, respectively. (**E**) Stau1 inhibits *in-vivo* PKR Ser/Thr phosphorylation in MH14 cells. Left panel: Cured MH14 cells were mock-transfected without HCV RNA (lane 1) or co-transfected HCV RNA with Stau1 and PKR overexpression plasmids (lanes 2–8). Lane 2, MH14 cells transfected with HCV RNA alone or co-transfected with empty vector alone. Lane 3, MH14 cells transfected with 1.5 μg pEGFP Stau1 plasmid (lane 4) or with 1.5 μg pEGFP-PKR plasmid without or with increasing concentration of pEGFP Stau1 plasmid (0.5, 1.0, 1.5 μg) (lanes 5–8). Transfected cells were grown for 48 h. Cells were lysed in the presence of phosphatase inhibitor cocktail. Cell lysates were normalized and immunoprecipitated using PKR antibody; then western blotted for phosphorylated PKR using specific serine-threonine-specific phospho-antibody. Cell lysates were western blotted for total PKR, Stau1 and actin. **Right panel**: Quantitation of WB bands of serine-threonine-phosphorylated PKR.

Since Stau1 stimulates HCV replication and promotes translation of viral proteins, we examined whether it also interacts with viral proteins. We also examined whether Stau1 interacts with PKR, which is involved in restricting the translation of HCV RNA by phosphorylation of initiator Met-tRNAf-binding factor eIF2α in the cells. PKR is an interferon-stimulated gene that is critical for cellular antiviral and antiproliferative responses. It has been implicated as a tumor suppressor because of its ability to phosphorylate eIF-2a (42,43). Since Stau1 promotes both viral replication and translation, we postulated that it may interact with components of the HCV replication complex as well as PKR. To ascertain this hypothesis, we performed Stau1-IP on benzonase treated cell lysates (50 μg) from JFH1 HCV-infected Huh7.5 cells, and western blotted for NS5B and PKR. We detected both viral protein NS5B and cell factor PKR co-immunoprecipitated with Stau1-IP (Figure 6A, top panel, lane 1). PKR-IP on the cell lysate also co-precipitated Stau1 (Figure 6A, bottom panel, lane 1). Using IP on purified recombinant proteins (200 ng each protein), we confirmed that Stau1 directly interacts with NS5B and PKR. Stau1-IP on a mixture of Stau1+NS5B co-precipitated NS5B (Figure 6B, lane 1, top panel). Reciprocal IPs on a mixture of PKR+Stau1 also coprecipitated each other (Figure 6B, lane 1, bottom panel). These results indicate that the interaction between Stau1 and PKR may modulate activation of the latter for promoting translation of viral proteins while the interaction between Stau1 and NS5B suggest that Stau1 may be a component of the HCV replication complex.

### Stau1 strongly inhibits the autophosphorylation of PKR

Since PKR and Stau1 co-immunoprecipitate each other, we examined whether Stau1 has any effect on the autophosphorylation of PKR, which is an essential step for its activation and dimerization (44). Using increasing concentrations of purified PKR (1-4μg) in a standard autophosphorylation assay (37,38), we found that recombinant PKR is efficiently autophosphorylated (Figure 6C; left panel, lanes 1–4). In the presence of increasing concentrations of Stau1, the autophosphorylation of PKR was strongly inhibited (Figure 6C; right panel, lanes 2–4). We observed two bands of phosphorylated recombinant PKR in the gel. We presumed that the recombinant PKR is a mixture of partially phosphorylated and unphosphorylated protein, and both are the substrate for autophosphorylation. This premise was confirmed by the disappearance of the upper band when recombinant PKR was treated with the calf intestine alkaline phosphatase (Figure 6D). These results clearly indicate that the interaction between PKR and Stau1 is required for strong inhibition of PKR activation in HCV-infected cells and that PKR inhibition is required for viral RNA translation in the cells through the use of cellular translational machinery.

### Stau1 inhibits *in-vivo* PKR Ser/Thr phosphorylation in MH14 cells

To confirm the effect of Stau1 on *in-vivo* autophosphorylation of PKR, we co-transfected cured MH14 cells devoid of HCV replicon with HCV RNA along with overexpression plasmids of Stau1 and PKR. Forty-eight hours later, PKR-IP was performed on cell lysates and western blotted for the level of Ser and Thr phosphorylation of PKR using specific phospho-(Ser/Thr)Phe antibody (Cat # 9631, Cell Signaling). We found that overexpression of PKR in the presence of HCV RNA replicons enhances PKR autophosphorylation by 3- to 4-fold (Figure 6E, lane 5, left panel) as compared to that in controls (Lane 2). However, in the presence of increasing concentrations of Stau1, autophosphorylation of PKR in PKR-overexpressing cells was strongly inhibited (Figure 6E, lanes 6–8, left panel). These results confirm that PKR activation in HCV-infected cells is strongly inhibited by Stau1.

### Like Stau1, PKR binds domain IIId on HCV 5′NTR

**Figure 7.**
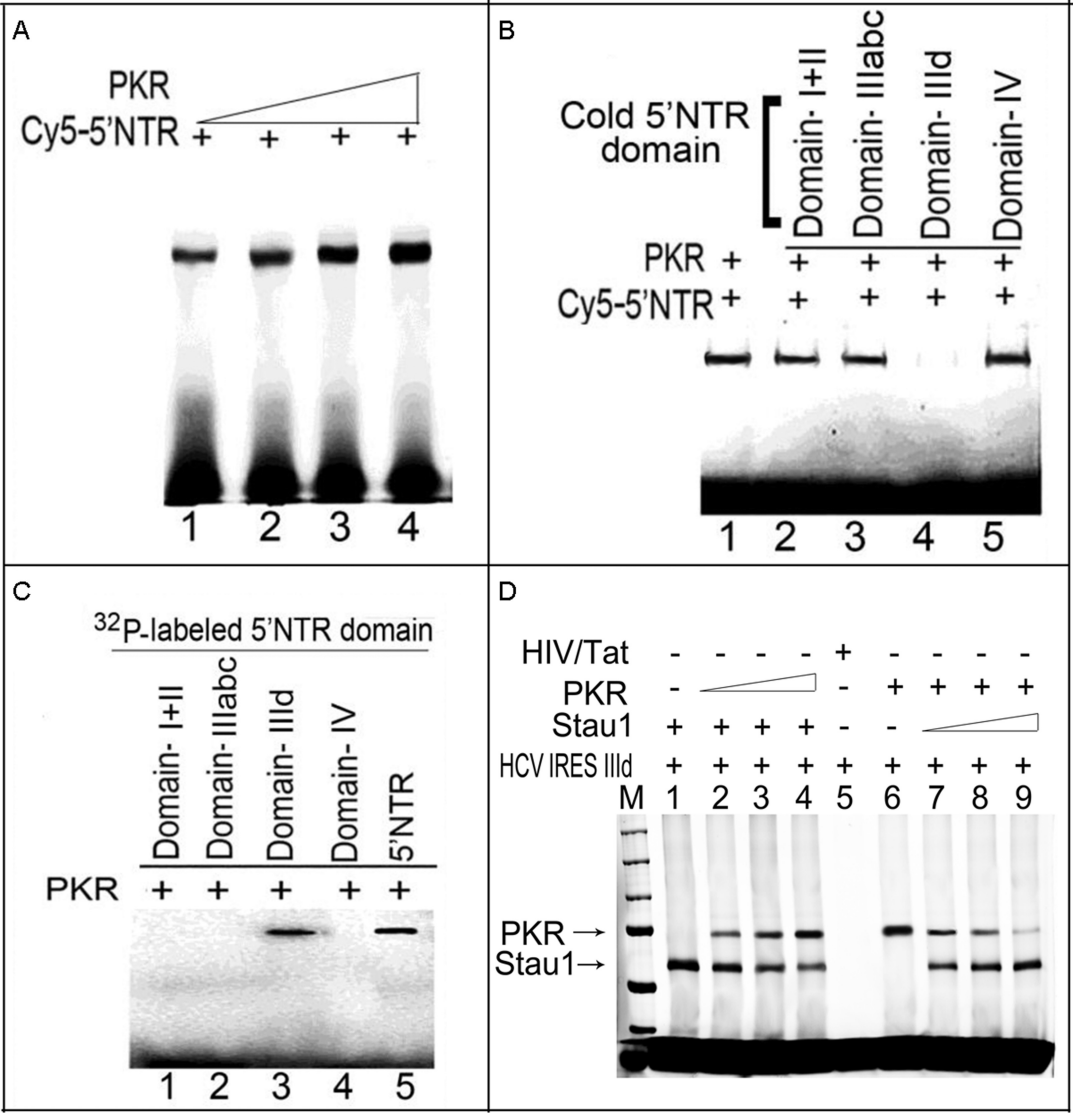
PKR binding to HCV 5′NTR is out-competed by HCV IRES domain IIId. (**A**) PKR binds to HCV 5′NTR. One picomole of Cy5-labeled HCV 5′NTR was incubated with increasing concentrations of purified PKR (0.75, 1.5, 3 and 6 pmol) (lanes 1–4). The samples were UV-irradiated, then treated with RNase A and resolved by SDS-PAGE. Crosslinked RNA–protein complexes were detected using a Typhoon scanner. (**B**) PKR competes specifically for domain IIId of the IRES region. Purified PKR was crosslinked with Cy5-labeled 5′NTR in the absence (lane 1) or presence (lanes 3–5) of unlabeled competitor RNA corresponding to domains I+II, IIIabc, IIId and IV. (**C**) PKR specifically binds to domain IIId of HCV IRES. Internally ^32^P-labeled *in-vitro*-transcribed RNA fragments corresponding to domains I+II, IIIabc, IIId and IV were crosslinked to PKR, treated with RNase A and resolved by SDS-PAGE. Only domain IIId crosslinked with PKR (lane 3). (**D**) Stau1 and PKR compete for the same binding site on HCV 5′NTR IIId region. We first incubated a fixed concentration (1 pmol) of Stau1 or PKR with 1 pmol *in-vitro*-transcribed Cy5-labeled domain IIId RNA on ice for 15 min and then supplemented with increasing concentration (1–3 pmol) of the competitor (PKR or Stau1) protein. After 20-min incubation on ice, the mixture was photocrosslinked, treated with RNase-A and resolved by SDS-PAGE. Lanes 1 and 6, respectively, represent binding of Stau1 and PKR to HCV IRES domain IIId RNA in the absence of competitor protein. Lanes 2–3 represents the competition of Stau1 binding to domain IIId RNA with PKR; Lanes 7–9 represent the competition of PKR binding to domain IIId RNA with Stau1.

Having found that PKR binds with HCV-5′NTR and suppresses viral translation (45), we examined whether PKR also selectively photo-crosslinks with domain IIId of the 5′NTR, as Stau1 does. As shown in Figure 7A, photo-crosslinking of Cy5-labeled HCV 5′NTR with PKR increased with increasing concentrations of PKR (Figure 7A, lanes 1–4). The crosslinking of Cy5-labeled HCV 5′NTR to PKR is selectively out-competed by cold RNA corresponding to domain IIId of HCV IRES (Figure 7B, lane 4). We confirmed that, like Stau1, PKR selectively binds to ^32^P-labeled domain IIId of HCV IRES (Figure 7C, lane 3). This indicates that Stau1-may compete with PKR for binding to the domain IIId region of HCV IRES and thus may block PKR-mediated inhibition of HCV translation.

#### Stau1 and PKR compete for the same binding site on HCV 5′NTR IIId region

Since PKR and Stau1 specifically bind to domain IIId of HCV IRES, we examined whether they compete for the same binding site. To test this, we first incubated a fixed concentration of Stau1 or PKR with Cy5 labeled domain IIId RNA and then supplemented with increasing concentration of the competitor protein (Figure 7D). We found that both Stau1 and PKR efficiently outcompeted each other for binding to domain IIId of HCV IRES. (Figure 7D, lanes 2–4 and 7–9). Since Domain III-V of HCV IRES is known to activate PKR (45,46), our finding suggests that competing out PKR for binding to HCV IRES may be one of the possible mechanism in which Stau1 may inhibit the activation of PKR and promotes HCV replication and translation.

### Stau1 enhances the *in-vitro* RdRp activity of NS5B

**Figure 8.**
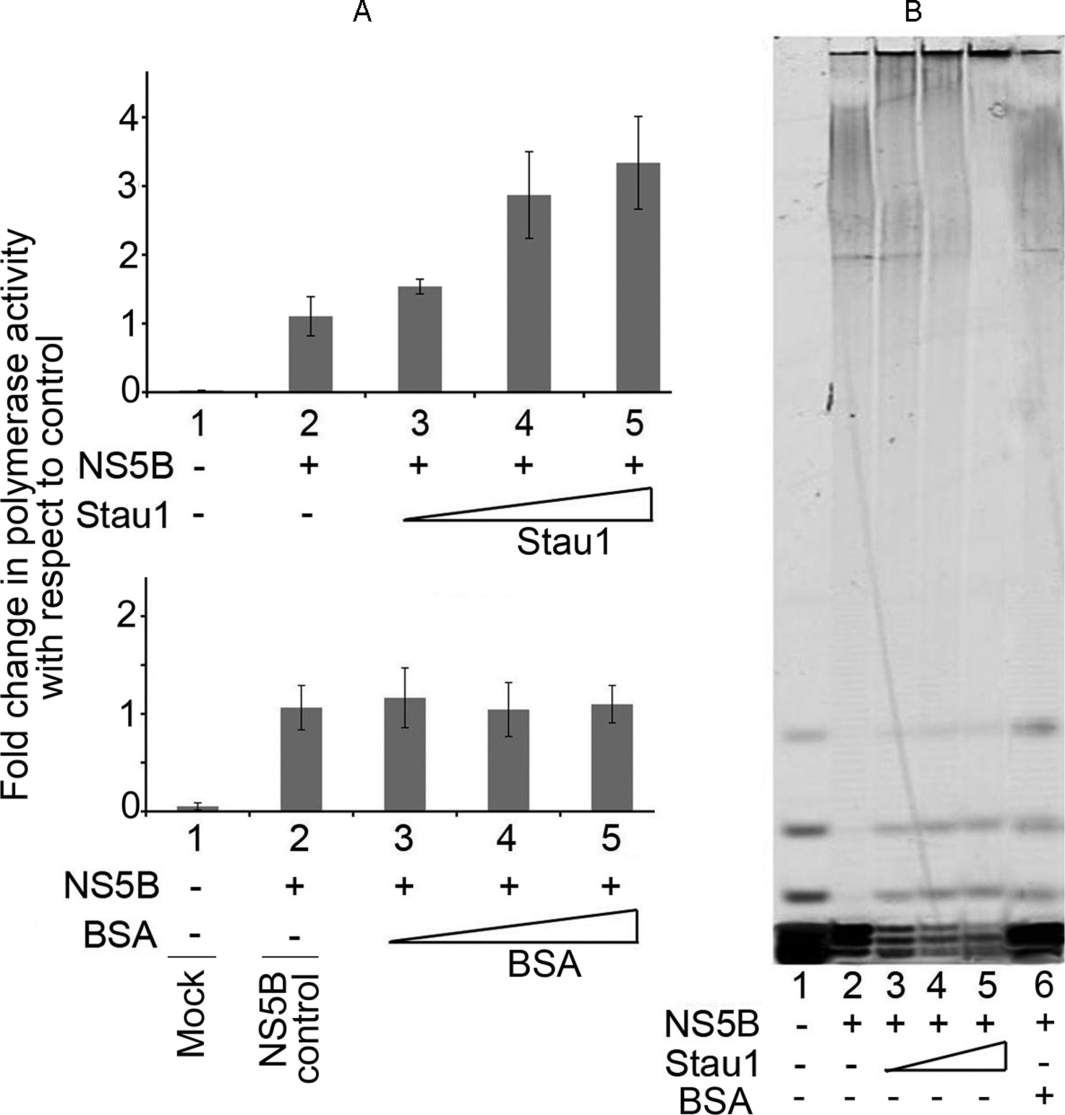
Effect of Stau1 on RdRp activity of HCV NS5B. (**A**) The RdRp activity of NS5B (100 nM) in the absence or presence of increasing concentrations of recombinant Stau1 (top panel) or bovine serum albumin (bottom panel) was determined using poly (rA)/dT_18_ as the template-primer and ^3^H-UTP as the substrate. Acid-precipitable radioactive reaction products were collected on Whatman GF/B filters and counted for radioactivity. The results are expressed as percent of control. Lane 1, blank; lane 2, NS5B alone; lanes 3–4, NS5B with increasing concentration (200–800 nM) of Stau1 or BSA (**B**) Gel analysis of primer extension product catalyzed by NS5B. We did a primer extension assay using poly (rA)/^Cy5^-dT_18_ as the template-primer and cold UTP as the substrate. Lane 1, input template-primer; lane 2, 100 nM NS5B alone; lanes 3–5, 100 nM NS5B in the presence 200, 400 and 800 nM of Stau1; lane 6, 100 nM NS5B in the presence of 800 nM BSA.

Since Stau1 co-immunoprecipitates NS5B and enhances endogenous HCV replication in cell-free replication lysate, we examined whether the *in-vitro* RdRp activity of NS5B is also influenced by the presence of purified recombinant Stau1. We examined the RdRp activity of NS5B in the presence and absence of Stau1 (Figure 8A). To do so, we first incubated NS5B with either Stau1 or bovine serum albumin (BSA) at a 1:1 molar ratio for 10 min on ice, then assayed for RdRp activity using homopolymeric rA/dT18 template-primer and ^3^H-UTP as the substrate. The acid precipitable reaction products were collected on Whatman GF/B filters and counted for radioactivity in a liquid scintillation counter. We found a 2-fold increase in the RdRp activity of NS5B in the presence of Stau1, and then confirmed this by gel analysis of the primer extension catalyzed by NS5B in the presence and absence of Stau1. We used Cy5-labeled oligo dT_18_ annealed with poly rA as the template-primer and cold UTP as the substrate. The extension products were resolved on an 8% denaturing polyacrylamide-urea sequencing gel and visualized by Typhoon imager. We found a similar stimulatory effect of Stau1 on the polymerase activity of NS5B (Figure 8B, lanes 2–5). To confirm whether enhanced activity of NS5B in the presence of Stau1 is not due to protein stabilizing effect, we also carried out reaction in the presence of BSA and found no change in the polymerase activity (Figure 8B, lane 6). These results imply that Stau1 may have a role in facilitating HCV replication.

### Co-localization of Stau1 with PKR and NS5B

**Figure 9.**
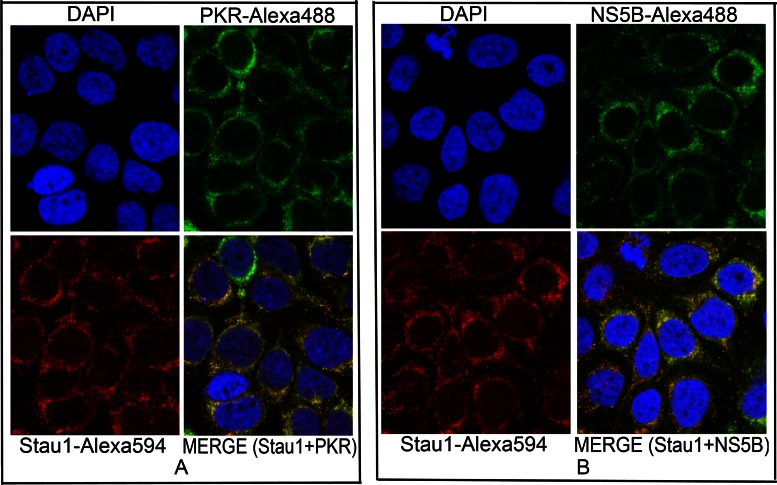
Co-localization of Stau1 with PKR and NS5B in HCV-infected Huh7.5 cells. (**A**) Co-localization of Stau1 and PKR. Cells grown on eight-chamber tissue-culture slides for 24 h were fixed, treated with mouse anti-PKR antibody, then with anti-mouse Alexa-488 labeled secondary antibody (green). The same set of cells were then treated with the anti-Stau1 antibody (rabbit) and anti-rabbit Alexa-594-labeled secondary antibody (red) (25). Cells were observed by Nikon confocal microscope (A1R) for localization of PKR and Stau1. Pictures were taken individually and merged using NIS-Element Viewer 4.20 software (Nikon). (**B**) Co-localization of Stau1 and NS5B. We used goat anti-NS5B antibody as the primary antibody and anti-goat Alexa-488 as the secondary antibody (green). Cells were observed by Nikon confocal microscope for localization of NS5B and Stau1. Pictures were taken individually and merged using NIS-Element Viewer 4.20 software. Stau1 colocalized with PKR and NS5B with an overlap coefficient of 0.88 and 0.95, respectively.

Stau1, being a cytoplasmic dsRNA binding protein, is mainly localized in the cytosol, which is also the site of HCV replication. We also found that Stau1-IP co-immunoprecipitates HCV viral protein NS5B from the cell lysate of HCV-infected Huh7.5 cells. Stau1 is also involved in the transfer and loading of HCV RNA to polysomes, which are associated with the endoplasmic reticulum, where the membranous web for HCV replication is also located. Since NS5B is the main component of the intracellular-membrane-associated replication complex (47), we examined whether Stau1 is also co-localized with the viral protein NS5B in MH14 cells containing replicating HCV subgenomic replicons. We also examined co-localization of Stau1 and PKR. We did immunofluorescence staining of Stau1 and NS5B, as well as Stau1 and PKR and examined their images by the confocal microscope. We found that Stau1 is co-localized with both PKR (Figure 9A) and NS5B (Figure 9B) in the cytoplasm. As earlier shown by others (9,11), we found Stau1 was mainly localized in the nuclear periphery, presumably in the endoplasmic reticulum (ER). We further found that Stau1 was colocalized with NS5B and PKR in the nuclear periphery with an overlap coefficient of 0.95 and 0.88, respectively.

## DISCUSSION

The 63-kDa Stau1 is a double-stranded RNA-binding protein having five dsRBDs. We found that Stau1 specifically binds to both the stem-loop domain IIId of HCV 5′NTR (Figure 2E and F) and variable stem-loop (VSL) region of 3′NTR (Figure 1 C and D). Mutagenesis studies have shown that the GGG triplet (nucleotides 266 through 268) of the apical stem-loop of domain IIId is essential for IRES activity (48) and that interaction of Stau1 with this domain may be crucial for translation of viral proteins. Interaction of Stau1 with 5′ NTR of mRNAs facilitates the translation of these mRNAs (17). The VSL region in 3′NTR is essential for efficient viral replication (49). Any deletion in the VSL region led to a partial loss of replication capacity (40). Stau1 interacting with the VSL region in 3′NTR may play a role in HCV replication. The fact that downregulation of Stau1 inhibits both HCV replication and translation (Figure 3A) indicates that Stau1 may be crucial in facilitating and establishing HCV infection.

dsRNA-binding proteins (DRBPs) residing in the cytoplasm regulate translation, dsRNA signaling events and host defense, whereas DRBPs in the nucleus, are involved in RNA interference (RNAi) and mRNA elongation, editing, stability, splicing and export (26). We have demonstrated that Stau1 is a cytoplasmic DRBP that physically interacts with interferon-induced PKR in the cytoplasm. The interferon-inducible PKR, also a cytoplasmic dsRBP, is a key factor in the host innate defense mechanism, displaying strong antiviral and antigrowth activities. PKR is activated by direct binding of dsRNA (50). The autophosphorylation of PKR leads to its activation, which, in turn, phosphorylates the alpha subunit of the eukaryotic translation initiation factor 2 (eIF2α) (42,43).

Phosphorylated eIF2α disrupts translational initiation of viral and cellular mRNAs. HCV protein synthesis requires the activity of eIF2α, which is strongly inhibited by PKR. Many viruses, including HCV, have developed mechanisms to inhibit PKR to prevent the shut-off of cellular protein synthesis, which would be detrimental to their replication (27). HCV-IRES and NS5A both interact with PKR and inhibit its autophosphorylation and activation (37,51). The viral protein, NS5A, interacts within the dimerization domain of PKR and disrupts its dimerization (51). We found strong inhibition of *in-vitro* autophosphorylation of PKR by Stau1 (Figure 6C, left panel), suggesting its possible involvement in preventing a PKR-mediated translational shutdown in cells. Since PKR physically interacts with Stau1 and displays similar binding specificity to domain IIId of the HCV-IRES (Figure 7A and B), it is possible that Stau1 modulates PKR activation by blocking its binding to domain IIId of HCV IRES. Elbarbary et al. have recently shown that binding of Stau1 to cellular mRNAs enhances their translation by precluding PKR binding, which prevents PKR activation, and phosphorylation of eIF2α resulting in repression of global cell translation (52).

Overexpression of Stau1 enhances both HCV replication and translation in Huh7.5 cells (Figure 3B). Stau1 is mainly localized to the rough endoplasmic reticulum (5) in the ribonucleoprotein complex, with ribosomes surrounding the nuclear periphery in mammalian cells (11). The RER dotted with ribosomes is the site of protein synthesis and folding. The site of HCV replication in the membranous web is also in the vicinity of RER. Stau1 participates in the formation of cytoplasmic RNA granules, which control localization and translation of specific cellular mRNAs (53–55). In this context, specific binding of Stau1 with domain IIId of HCV IRES (Figure 2E and F) may facilitate the transport of viral RNA onto the translation machinery. Polysome fractionation indicated that Stau1 is required to facilitate transport of HCV RNA to the polysomes. The overexpression of Stau1 significantly enhances the transport of viral RNA in the polysomes fractions while, in the absence of Stau1, it remained in the monosome fractions (Figure 5A, B and C). These observations indicate that Stau1 is required for the docking of HCV RNA onto the polysomes for efficient translation and synthesis of viral proteins. A previous study reported that Stau1 is associated with 60S ribosomal subunits (56) and is involved in de-repression of Oscar mRNAs translation (57).

Interestingly, Stau1-IP co-precipitates HCV replicase (NS5B) from the cell lysate of HCV-infected cells. We confirmed the direct interaction of Stau1 with NS5B by immunoprecipitation on a mixture of purified recombinant proteins of Stau1-NS5B, as well as immunofluorescent staining of Stau1 and NS5B, which showed that Stau1 was co-localized with NS5B. An earlier report showed co-localization of Stau1 with another viral protein, HCV NS3, in the nuclear periphery of HCV-infected cells (9).

Stau1 is known to be involved in the splicing, transport, localization, translation and stability of cellular mRNAs (10,17,19). It is present in cytoplasmic stress granules harboring abortive translation initiation complexes, which contain inactive phosphorylated eIF2α as one of the constituents (55). It has been proposed that Stau1 may be involved in recovery from cellular stress by stabilizing polysomes and that it may facilitate the dissolution of stress granules (55). In this context, our finding that Stau1 inhibits autophosphorylation and inactivates PKR, which is responsible for inactivation and phosphorylation of eIF2α, is significant. We speculate that the association of Stau1 with stress granules may trigger dephosphorylation of eIF2α, which may facilitate dissolution of the stress granules and recovery from cellular stress.

**Figure 10.**
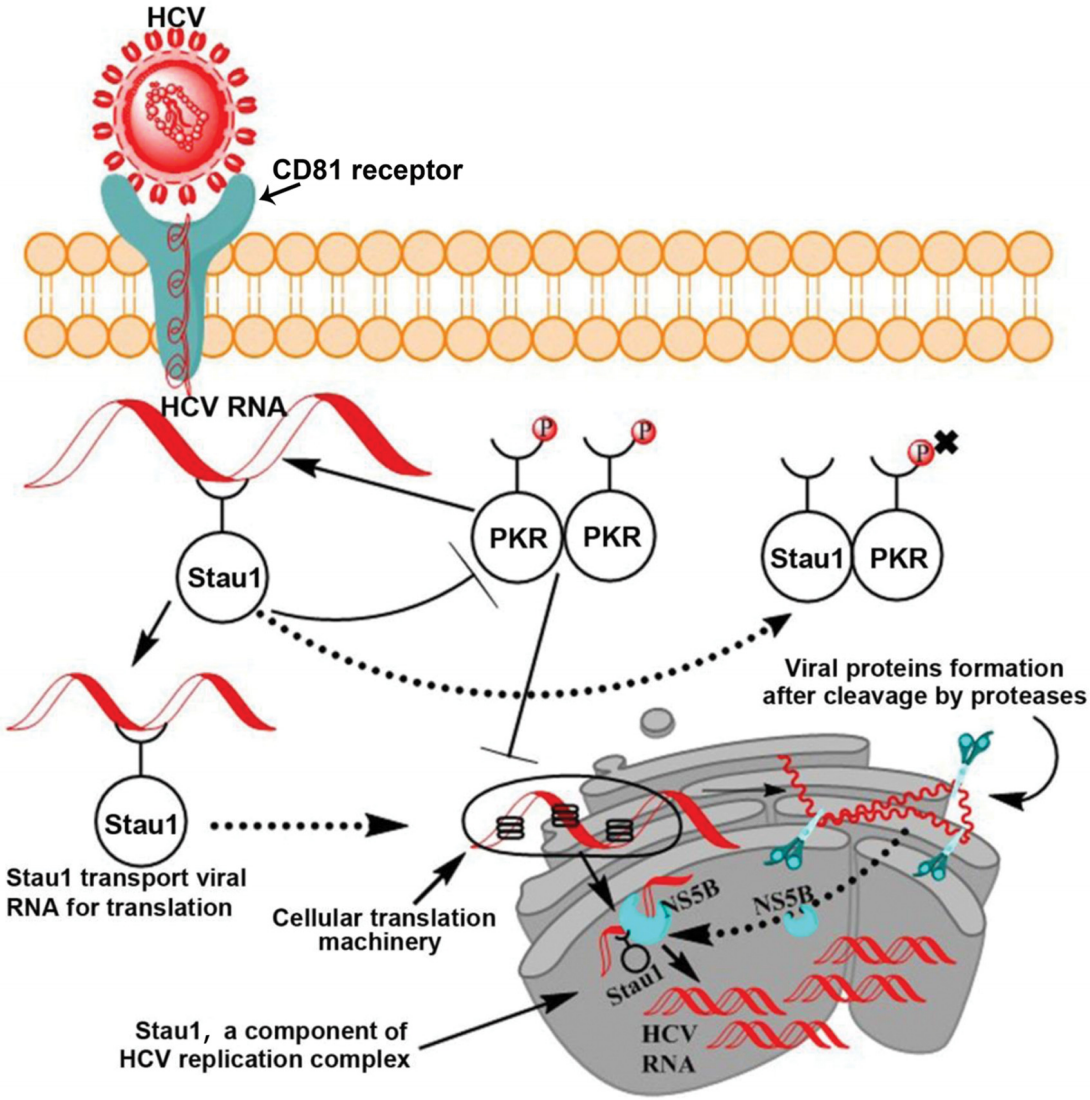
Schematic model of Stau1 interaction and its implication on HCV replication/translation. Following the entry of viral RNA into the cell, activation of PKR is blocked by Stau1, either by preventing dimerization of PKR by inhibiting its autophosphorylation or by forming an inactive heterodimer consisting of PKR: Stau1 heterodimer. Stau1 facilitates the transports of viral RNA to the ribosomes/RER where viral proteins are synthesized and folded. Stau1 then transport viral RNA to the newly formed membranous web in the vicinity of RER for replication. Since NS5B, NS5A, NS3 and HCVRNA are constituents of the HCV replication complex, and Stau1 interacting with these constituents could as well be part of the replication complex, besides facilitating transport of HCV RNA at the site of translation/replication.

Based on these results, a schematic model of Stau1 interaction is shown in Figure 10. In this model, we propose that, following the entry of viral RNA into the cell, activation of PKR may be blocked by Stau1 by (i) outcompeting PKR binding to HCV IRES; (ii) directly interacting with PKR and inhibiting its autophosphorylation and dimerization or (iii) forming an inactive PKR: Stau1 heterodimer. PKR has been shown to form heterodimer with many unrelated ds-RNA binding proteins including vaccinia virus E3L (58), TRBP (59), NFAR (60), SPNR (61) and PACT (62). Dimerization of PKR with other ds-RNA binding proteins has been shown to be mediated by their respective ds-RNA-binding motifs (59). The dsRNA-binding motifs of a number of DRBPs including Stau1 have been shown to be homologous with those of PKR (63). The interference with homodimer formation of PKR through the formation of inactive heterodimers is one of the potential mechanisms for inhibiting kinase activity of PKR (61). Since we have shown that Stau1 physically interacts with PKR and inhibits its autophosphorylation and activation, it is most likely that their interaction is via their dsRNA binding motifs.

Since Stau1 is located in the in the nuclear periphery in the ER (9,11) and is essential for loading HCV RNA onto the polysomes (Figure 5), we propose that Stau1 transports viral RNA to the ribosomes and on to the RER for synthesis and folding of viral proteins. Following translation, viral RNA is then transported onto the newly formed membranous web in the vicinity of RER for replication of viral RNA by HCV replication complex. Since NS5B, NS5A, NS3 and HCVRNA are constituents of the HCV replication complex, the interaction of Stau1 with these constituents could as well be part of the replication complex, besides facilitating transport of HCV RNA at the site of translation or replication. Stau1 also influences the replication and infectivity of Influenza virus via its interaction with viral protein, NS1 (25,64). It plays an important role in HIV-1 assembly, replication, translation and generation of infectious virions particles (65–67). A similar role for Stau1 can be envisaged in the promotion of HCV replication and production of virion particles.
